# The first report on *Hepatozoon canis* in dogs and wolves in Poland: clinical and epidemiological features

**DOI:** 10.1186/s13071-023-05928-5

**Published:** 2023-09-04

**Authors:** Katarzyna Tołkacz, Milena Kretschmer, Sabina Nowak, Robert W. Mysłajek, Mustafa Alsarraf, Dagmara Wężyk, Anna Bajer

**Affiliations:** 1 Institute of Biochemistry and Biophysics, Polish Academy of Sciences, 5A Pawińskiego Str, 02-106 Warsaw, Poland; 2 Vetlab Sp. Z O. O., Veterinary Diagnostic Laboratory, Wodzisławska Str 6, 52-017 Wrocław, Poland; 3 Department of Ecology, Institute of Functional Biology and Ecology, Faculty of Biology, Biological and Chemical Research Centre, University of Warsaw, Warsaw, Poland; 4 Department of Eco-Epidemiology of Parasitic Diseases, Institute of Developmental Biology and Biomedical Sciences, Faculty of Biology, University of Warsaw, Warsaw, Poland

**Keywords:** *Canis lupus familiaris*, *Hepatozoon canis*, Hepatozoonosis, Poland, PCR, Ticks, Wolves

## Abstract

**Background:**

Canine hepatozoonosis caused by *Hepatozoon canis* is a common infection in dogs, with frequent case reports from the Mediterranean region and more recently from several Central European countries, such as Hungary and Germany. Despite the high prevalence of *H. canis* in red foxes, no infections have been reported to date in dogs in Poland. We describe here the first autochthonous cases of *H. canis* infection in dogs, including their clinical features, and report the prevalence of *H. canis* in grey wolves from different regions of Poland.

**Methods:**

Thin smears prepared from blood samples collected from dogs were evaluated by microscopic examination. A total of 60 wolves and 47 dogs were tested. Infections were confirmed by PCR and sequencing.

**Results:**

Gamonts of *H. canis* were found in > 50% of the neutrophils of two dogs and in < 10% of the neutrophils in another five dogs. Molecular typing by PCR sequencing of the *18S* ribosomal RNA gene fragment confirmed infections in 11 dogs from different regions of Poland, in 2.7% of dogs attending veterinary practices in central Poland and in 35% of wolves from various geographical regions of Poland. Clinical features manifested mostly in older dogs, and the most common signs were anaemia and apathy. Young dogs usually remained asymptomatic.

**Conclusions:**

This is the first report of *H. canis* infection in dogs and wolves in Poland. Although the exact vector of the parasite is not known, veterinary practitioners should be aware of this new parasitosis and should consider appropriate diagnostics to confirm/exclude this infection. Further studies are needed to understand the transmission routes of *H. canis* in domestic and wild canids in Poland.

**Graphical Abstract:**

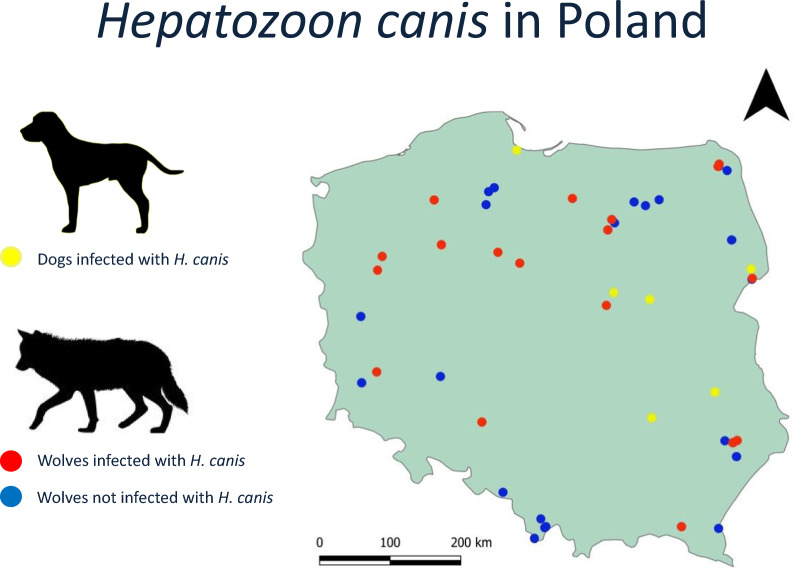

## Background

*Hepatozoon canis* is an apicomplexan parasite vectored mainly by the brown dog tick *Rhipicephalus sanguineus *sensu lato (*R. sanguineus *s.l.). The presentation of *H. canis* infection may vary from asymptomatic in apparently healthy dogs to severe and life-threatening clinical signs, with affected animals displaying extreme lethargy, cachexia and anaemia [1, 2]. Vertical transmission of *H. canis* from an infected female to its pups has also been demonstrated [3].

*Hepatozoon canis* infection in dogs was initially described by S.P. James in India in 1905 [4]. Hepatozoonosis is a common disease in dogs in the Mediterranean area of Europe and more recently was also reported in both South and North America [1, 2, 5–7]. The expansion of *H. canis* to Central Europe has been confirmed recently, likely due to the northwards expansion of the geographical range of *R. sanguineus* with climate change and global warming [2, 8, 9]. The first cases of *H. canis* infection in Central Europe were reported in dogs in Hungary [10], Ukraine [11], the Czech Republic [12] and Germany [13], despite the absence of established *R. sanguineus* populations. Imported *H. canis* cases were also recently identified in the UK [14].

A surprisingly high prevalence of *H. canis* was also identified in free-living carnivores in Central Europe, including red foxes [13, 15–21], golden jackals [18, 22] and grey wolves [23]. In Poland, several previous studies have reported the absence of *Hepatozoon* in different groups of dogs screened for the disease [24, 25]. However, this parasite has been reported in red foxes in Poland, with an increasing prevalence in the last 10 years [16, 19].

The aim of this study was to describe the first autochthonous cases of *H. canis* infections in dogs, including their clinical features, and to determine the prevalence of *H. canis* in grey wolves from different regions of Poland. Molecular methods were used to compare the genetic identity of parasites from dogs and wolves.

## Methods

### Ethics approval and consent to participate

**Fig. 1:**
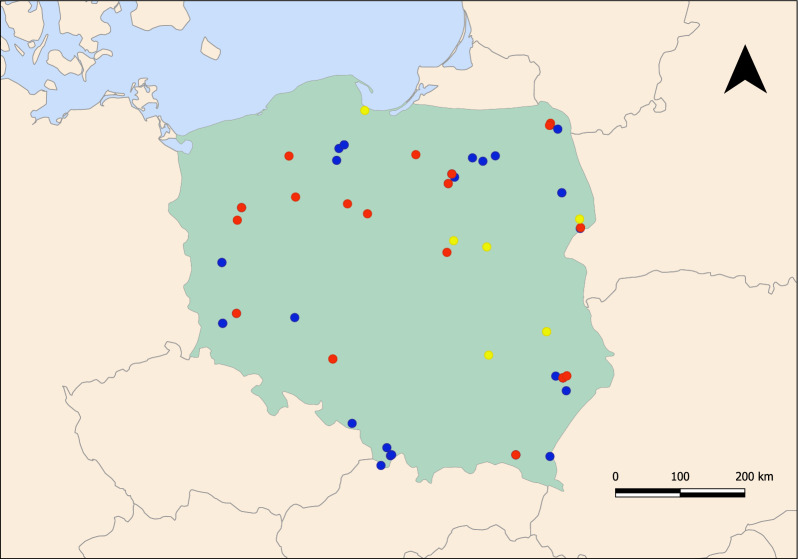
Map of Poland presenting locations where dogs and wolves were found. Collection sites for dogs and wolves. Yellow dots represent the collection site of the infected dogs, blue dots represent the collection site of noninfected wolves and red dots represent *Hepatozoon* *canis*–positive wolf samples. The territorial extent of Poland is highlighted by the green-shaded area, with neighbouring countries depicted by orange-shaded areas. The grey lines represent international borders, and the Baltic Sea is represented by the light blue-shaded area

The study was carried out on blood samples provided voluntarily by dog owners; thus, no ethical approval/licence was required for this study (as per Resolution on the protection of animals used for scientific or educational purposes, 15th January 2015 [Dz. U. 2015 position 266] Chapter 1, Paragraph 1.2.1). The owners of dogs involved in this study were informed of the aim of the study and provided oral consent and contact information to obtain the results of testing. For the epidemiological investigation, we used samples from healthy dogs and dogs suspected of having babesiosis (*n* = 37) that were collected in 2020 in Tłuszcz, Mazovia, central Poland [26]. Another eight samples (cases 1–8) were obtained from the veterinary laboratory Vetlab in 2021–2022 (Fig. 1). These samples were collected from dogs admitted to various veterinary practices for routine check-ups or because of poor health conditions. The remaining two samples (cases 10–11) were obtained in 2022 from two elderly dogs from Błedowo (Mazovia, central Poland) suffering from recurring anaemia of unknown origin. These two samples were also checked by PCR for *Babesia* and *Dirofilaria* spp. infection as described previously [26, 27].

### Wolf samples

Blood and tissue (*n* = 57) and faecal (*n* = 3) sampling of wolves from several locations in Poland was conducted (Fig. 1). Blood and tissue samples from wolves were collected between 2016 and 2022 from individual wolves killed in traffic accidents, illegally shot or snared, found dead due to diseases and other natural causes, captured for telemetry studies or delivered to wildlife rehabilitation centres due to malnutrition and health problems or from vaginal discharges of adult breeding females tracked during mating seasons [28]. Vaginal discharge blood on fresh snow and faecal samples were found during the tracking of wolves. No animals were specifically killed for this study. The Polish General Directorate approved the collection of samples under Environmental Protection DZP-WG.6401.08.1.2017.bp. Permission for wolf capture and handling was granted by the Polish Ministry of Environment for field work conducted within the Roztocze National Park (DOP-WPN.286.309.2018.MD) and the Regional Directorate for Environmental Protection in Lublin for field work outside the national park (WPN.6401.18.2020.KC). All procedures were approved by the First Warsaw Local Ethics Committee for Animal Experimentation in Warsaw (ethical licence numbers: 759/2018, 977/2020) according to the principles governing the experimental conditions and care of animals required by the European Union and the Polish Law on Animal Protection.

### Detection of *H. canis* infection in wolves and dogs

DNA was extracted from blood or tissue samples from 47 dogs and 57 wolves following the manufacturer’s protocol for DNA extraction (DNAeasy Blood & Tissue Kit; Qiagen, Hilden, Germany). In addition, three faecal samples were collected into 50-ml tubes with 96% ethanol. DNA extraction from faeces was performed with a commercial kit (Exgene™ Stool DNA mini; GeneAll, Seoul, Korea) following the manufacturer’s protocol.

In one case, a methanol-fixed blood smear (not stained) from a dog diagnosed as positive for *Hepatozoon* sp. by microscopy (case 1) was used for DNA extraction as the only available source of the DNA. In this case, the smear was first cleaned with a methanol swab and then, 200 µl of lysis buffer (AL) (DNAeasy Blood & Tissue kit; Qiagen)—enough to cover the whole blood surface—was pipetted onto the smear. The smear was left to soak for 1.5 h in humid conditions, and then the blood was gently removed from the slide using a sterile disposable scalpel and pipetted into a 2-ml Eppendorf tube, following which 20 µl of proteinase K was added. The sample was incubated at 56 °C for 10 min and then processed further following the manufacturer’s protocol for DNA extraction from blood (DNAeasy Blood & Tissue kit; Qiagen). Genomic DNA was used in PCR amplification and then stored at − 20 °C.

All cases of *Hepatozoon* infection in dogs were diagnosed based on microscopic examination of Wright-Giemsa-stained blood smears and PCR testing.

PCR amplification and sequencing of a 666-bp fragment of the *18S* ribosomal RNA (rRNA) gene were performed for molecular identification of the *Hepatozoon* species, following a modified protocol described by Alsarraf et al. [29]. The forward primer (Hep1: 5′-CGCGAAATTACCCAATT-3′) and the reverse primer (Hep2: 5′-CAGACCGGTTACTTTYAGCAG-3′) were used in the PCR assays [30].

The positive control for the PCR assays consisted of DNA of *H. erhardovae* from rodents [31], and negative controls consisted of sterile water in the absence of template DNA. Nineteen PCR products (10 from dogs, 9 from wolves) were sequenced in both directions by a private company (Genomed S.A., Warsaw, Poland). Both reads were aligned and edited to form a consensus sequence using the BioEdit 7.2 tool [32]. The consensus sequence was compared with sequences deposited in the GenBank database (http://www.ncbi.nlm.nih.gov/genbank/). The phylogenetic analyses, including our sequences and sequences of *Hepatozoon* spp. deposited in the GenBank database, were conducted in MEGA v. 11 [33, 34]. The evolutionary model was chosen according to the data and bootstrapped over 1000 randomly generated sample trees. The maximum likelihood method was used for tree construction.

The results are presented as percentages with 95% confidence limits (CLs) in parentheses, calculated with bespoke software based on the tables of Rohlf and Sokal (W.H. Freeman and Co., New York; 1995), courtesy of F.S. Gilbert and J.M. Behnke from the University of Nottingham, UK. For comparison between wolf sexes and age groups, an unpaired two-tailed Student’s t-test was used.

### Case reports of infected dogs

#### Case 1, from Ostrowiec Świętokrzyski

An approximately 12-year-old male mixed-breed dog was admitted to the veterinary clinic in April 2021 in generally poor physical condition. His previous clinical history was unknown as the dog was found abandoned in a forest near the town Ostrowiec Świętokrzyski in southeastern Poland. Infesting ticks were removed without identifying their species. The animal presented a shaky gait (ataxia) and sunken eyes and was lethargic and malnourished (Table 1). Laboratory findings included nonregenerative anaemia, monocytosis and mild thrombocytosis (Table 2). Hyperproteinaemia with hyperglobulinaemia and increased alkaline phosphatase activity (ALP) were the main findings in the biochemical parameters. Magnetic resonance imaging of the head and neck showed inflammatory changes in the cerebellum and spinal cord.

**Table 1:** Cases of *Hepatozoon canis* infection in dogs from Poland

Dogs: *H. canis*-infected cases	Location	Date	Age	Clinical features	Co-infections
Case 1	Ostrowiec Świętokrzyski (SE Poland)	April 2021	10 years	Dog was lethargic and malnourished with shaky gait (ataxia), sunken eyes, increased alkaline phosphatase activity and inflammatory changes in the cerebellum and spinal cord	*Anaplasma* spp.; *Ehrlichia* spp.; *Toxoplasma gondii*
Case 2	Świdnik/Lublin	May/June 2022	12 years	Unknown	*Dirofilaria repens*
Case 3	Świdnik/Lublin	May/June 2022	2.5 years	Asymptomatic	*Dirofilaria repens*
Case 4	Świdnik/Lublin	May/June 2022	5 years	Asymptomatic	*Dirofilaria repens*
Case 5	Białystok-Hajnówka	May 2022	12–15 weeks	Weakness, lethargy, inappetence, general lymphadenopathy, tissue oedema and oozing, crusted skin lesions, especially of the nose and perianal region	*Babesia vulpes*
Case 6	Białystok-Hajnówka	May 2022	12–15 weeks	Asymptomatic	Unknown
Case 7	Gdańsk	June 2022	3.5 years	Asymptomatic	Unknown
Case 8	Białystok-Hajnówka	May 2022	12–15 weeks	Asymptomatic	Unknown
Case 9	Tłuszcz	October 2020	10 years	Symptoms resembling that of babesiosis in treating veterinarian's opinion	Excluded *Babesia canis* infection
Case 10	Błedowo	October 2022	At least 10 years	Generally poor conditions and prolonged anaemia, prolonged weakness and cachexia	Excluded *Babesia*, and *Dirofilaria* spp. infections
Case 11	Błedowo	October 2022	At least 10 years	Generally poor conditions and prolonged anaemia	Excluded *Babesia,* and *Diroilaria* spp. infections

**Table 2:** Values of the haemato-biochemical analyses from cases 1–6 and 9–10

Parameters	Case 1^a^	Case 2	Case 3	Case 4	Case 5^b^	Case 6	Case 9	Case 10^a^	Units^c^	Normal range
RBC	3.58↓	4.05↓	8.5	9.28↑	4.51↓	6.43	6.46	1.84↓	T/l	5.5–8.50
Thrombocytes	96↓	179	281	175	179	153	166	31↓	G/l	150,0–500,0
WBC	10.2	21.3↑	7.69	14.1↑	22.9↑	7.86	7.85	6.7	G/l	6.0–12.0
Neutrophils	5.1	18.2↑	3.56	7.43	17.5↑	4.21	4.46	0.1↓	G/l	3.0–9.0
Monocytes	1.79↑	1.62↑	0.56	0.81	2.8↑	0.49	0.98↑	0.6	G/l	0.150–0.850
Total protein	81.4↑	94.7↑	Nd	Nd	55.9	53.4↓	68.3	Nd	g/l	54.0–75.0
Globulin	56↑	64.9↑	Nd	Nd	33.8	25.3	46.6↑	Nd	g/l	18.0–45.0
Albumin	25.4	29.8	Nd	Nd	22.1	28.1	27.7	Nd	g/l	25.0–44.0
AST	Nd	100↑	Nd	Nd	27.8	47.7	32.4	28.0	IU/l	1.0–76.0
ALT	Nd	1036.5↑	155.6↑	70.5	14.8	34	49.3	32.0	IU/l	1.0–80.0
Parasitaemia	> 50%	> 50%	< 10%	< 10%	< 10%	< 10%	0%	0%	%	–

Vertical arrows indicate levels higher (↑) or lower (↓) than the normal range

*ALT* alanine aminotransferase, *AST* Aspartate aminotransferase,* Nd* not determined RBC red blood cell,* WBC* white blood cell

^a^Dog with cachexia

^b^Dog with *Babesia vulpes* co-infection

^c^G, Giga (10^9^); IU, international units; T, terra (10^12^)

#### Cases 2, 3 and 4 from Świdnik/Lublin

Three mixed-breed dogs were routinely examined during a first visit to a veterinary clinic in May and June 2022. All three dogs were housed together at the same location during that time, but they were originally stray dogs found in the city or in the suburbs. Blood and serum samples for haematology and biochemistry tests were collected from each dog during the examination. Clinical information on case 2 was not available, and neither of the other two dogs (cases 3 and 4) showed apparent clinical signs at the time of testing. However, all three dogs demonstrated haematological and biochemical alterations (Table 2). The oldest patient (case 2) presented the most pronounced haematological abnormalities, such as erythropaenia, leucocytosis with neutrophilia and monocytosis. Serum biochemistry showed an increased aspartate aminotransferase (AST) and alanine aminotransferase (ALT) activity, marked hyperproteinaemia, hyperglobulinaemia and low albumin to globulin (A/G) ratio (Table 2). Serum protein electrophoresis revealed polyclonal hypergammaglubulinaemia. Laboratory alterations in the other two dogs included mild leucocytosis, eosinophilia and lymphocytosis; elevated ALT was also observed in one dog (case 3; Table 2).

#### Cases 5, 6 and 8 from Białystok-Hajnówka

*Hepatozoon*-positive cases were identified in three juvenile dogs, approximately 12–15 weeks of age, from a farm in eastern Poland in Białystok-Hajnówka. The animals were kept in the barn where they had been born, but removed from their owner by an animal rescue foundation in May 2022.

One of the patients (case 5) demonstrated weakness, lethargy, inappetence, general lymphadenopathy, tissue oedema and oozing and crusted skin lesions, especially of the nose and perianal region (Table 1). The results of the blood and serum laboratory tests showed mild anaemia, leucocytosis with neutrophilia and monocytosis and slight hypoalbuminaemia (Table 2). The results of a canine rapid tick-borne disease test (CaniV-4; Vetexpert, Łomianki, Poland) and parvovirus test (rapid immunochromatographic antigen test) were negative, as were the results of an indirect semiquantitative immunofluorescence test against specific immunoglobulin G (IgG) antibodies against *Neospora caninum* (IFT, Megacor, Austria). PCR test results for *Toxoplasma* spp., *Anaplasma* spp. and *Ehrlichia* spp. (Synlab, Munich, Germany) were also negative. However, the puppy was positive for *Babesia vulpes* in the PCR assay, so imidocarb treatment was implemented immediately.

Case 6 was asymptomatic without significant laboratory alterations (Table 2). Mild low haematocrit (HCT) and haemoglobin (HGB) levels were noted.

No information on the blood parameters of the third puppy (case 8) was available, apart from it being asymptomatic.

#### Case 7 from Gdańsk

A 3.5-year-old female dog was brought to a local veterinary practice for a routine visit in June 2022 where a screening laboratory panel was performed. No relevant haematological abnormalities were detected (Table 2). The history of the patient was not clear, but it was known that it spent most of its life in the Gdańsk city area, without any movement outside of the region. The dog was taken from an animal shelter in Tczew as a 6-month-old puppy, so its previous location was unknown.

#### Case 9 from Tłuszcz

A 10-year-old mixed-breed male was brought to a local veterinary practice in October 2020 with a clinical presentation resembling that of babesiosis. Despite the signs suggesting *Babesia* infection (inappetence, general weakness), the dog tested negative for *Babesia canis* in both a Wright-Giemsa-stained blood smear and PCR tests and presented only with minor haematological alterations (Table 2). Follow-up information on this patient was not available.

#### Cases 10 and 11 from Błędowo

These two elderly rural dogs (> 10 years) underwent PCR screening for *Babesia*, *Hepatozoon* and *Dirofilaria* spp. due to generally poor conditions and prolonged anaemia of unknown origin. The first dog (case 10) suffered prolonged weakness and cachexia (Table 2). PCR test results for *Babesia* and *Dirofilaria* were negative. No additional clinical data were available for these dogs.

## Results

*Hepatozoon canis* infection was found in 11 dogs and 21 wolves from different regions of Poland (Fig. 1).

### Dogs

**Fig. 2:**
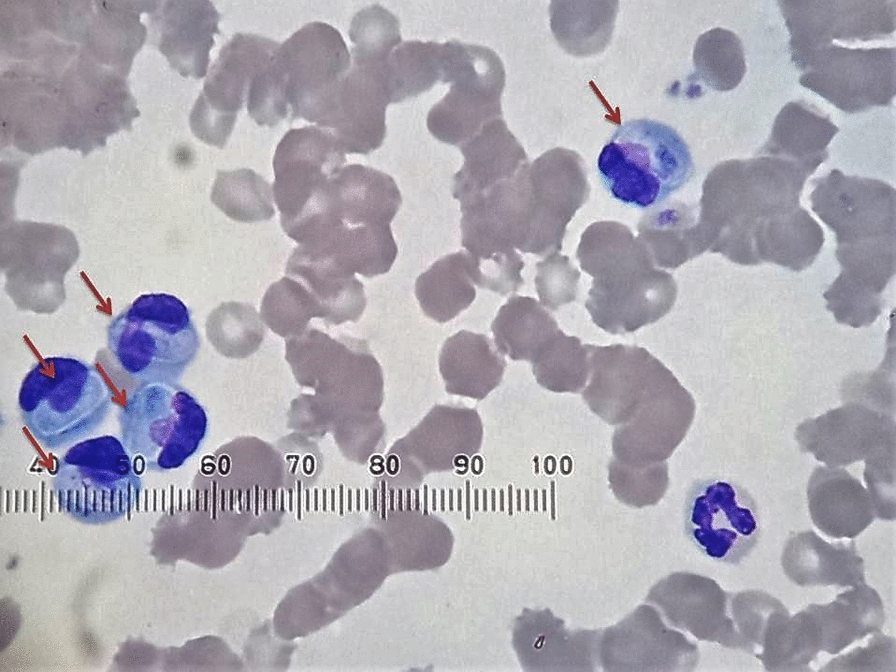
Gamonts (red arrows) of *Hepatozoon canis* in neutrophils in the peripheral blood of the dog (case 1)

In April 2021, the first reported case of *H. canis* infection was identified in samples sent to the Vetlab diagnostic laboratory (case 1). The examination of the blood smear stained using the Wright-Giemsa method showed numerous *Hepatozoon* sp. gamonts in neutrophils, and a subsequent PCR test confirmed *H. canis* infection (Fig. 2). In addition, positive serological test results were obtained for *Anaplasma* spp. and *Ehrlichia* spp. The patient also tested positive for IgM and IgG antibodies against *Toxoplasma gondii* (Table 1). Treatment with imidocarb dipropionate (repeated twice within 14 days) and doxycycline was implemented. Unfortunately, the dog was euthanised due to the lack of response to the treatment and the progressive worsening of clinical signs.

In the next three cases (2, 3 and 4; from Świdnik/Lublin), microscopic examination of Wright-Giemsa-stained blood smears allowed the detection of intracellular *H. canis* gamonts in neutrophils. The oldest dog (case 2), approximately 12 years of age, showed the highest parasite load, with up to 50% of neutrophils infected. Laboratory findings in that dog were similar to those reported in case 1, which was the first reported *H. canis* infection (April 2021). In the other two dogs (approximately 2.5 and 5 years of age, respectively), parasites were not observed in > 10% of the leukocytes. Co-infection with microfilariae was found in all three cases (Table 2). *Hapatozoon canis* infection was confirmed by PCR in all blood samples from these three dogs. Microfilariae were identified as *Dirofilaria repens* by PCR in the sample of one infected dog (Vetlab). Two patients with lower levels of parasitaemia were treated with oral doxycycline (10 mg/kg) for 4–6 weeks and spot-on imidacloprid once a month (dosage adapted to the weight of animal). A first posttreatment control 2 months after starting the therapy was negative for *H. canis* in both cases, based on microscopic examination of the blood smear and PCR testing.

Three young dogs housed together were confirmed to be infected with *H. canis* (cases 5, 6 and 8).

In the first puppy (case 5), despite the PCR results indicating positivity for both *H. canis* and *B. vulpes*, *Hepatozoon* gamonts were not detectable during microscopic examination of the Wright-Giemsa-stained blood smear and there were no visible *B. vulpes* merozoites (Table 1). A follow-up blood count and blood smear examination were performed 24 h after the administration of imidocarb to monitor haematological changes, revealing an additional finding, namely the presence of single *Hepatozoon* gamont in the cytoplasm of a few neutrophils.

In the second puppy (case 6), very low *Hepatozoon* parasitaemia was an incidental finding; however, infection was confirmed by a positive PCR test result. The treatment of those two dogs (cases 5 and 6) began with oral doxycycline (10 mg/kg/day) for 21 days, followed with imidocarb subcutaneously administered at 2-week intervals. The results of follow-up PCR blood tests were positive, indicating an ongoing *Hepatozoon* infection for at least 3 months post-treatment, although the haematological changes and clinical signs of the dog most affected (case 5) resolved.

*Hepatozoon canis* infection of the third, clinically asymptomatic puppy (case 8) was confirmed by PCR and sequencing.

Similarly, despite being asymptomatic, infection of an adult dog from Gdańsk (case 7) was confirmed by PCR (Table 1). Wright-Giemsa-stained blood smear examination also revealed low parasitaemia of *Hepatozoon* (Table 2). Despite no clinical signs of hepatozoonosis in this case, treatment was started (Table 2). Follow-up information on this patient’s treatment and current status was not available.

As the number of detected cases increased, we decided to check for the presence of *H. canis* in samples originating from dogs attending a small veterinary practice in central Poland in 2020. Examinations of these samples resulted in the identification of additional positive cases (chronologically reported after the first case) among dogs suspected of having babesiosis (case 9; Tables 1, 2).

Finally, two additional cases were identified in elderly dogs with chronic anaemia of unknown origin from another veterinary practice in central Poland in autumn 2022 (cases 10 and 11; Tables 1, 2). Both dogs tested positive in the PCR assays for *Hepatozoon*, with no gamonts detectable in Giemsa-stained blood smears.

**Fig. 3:**
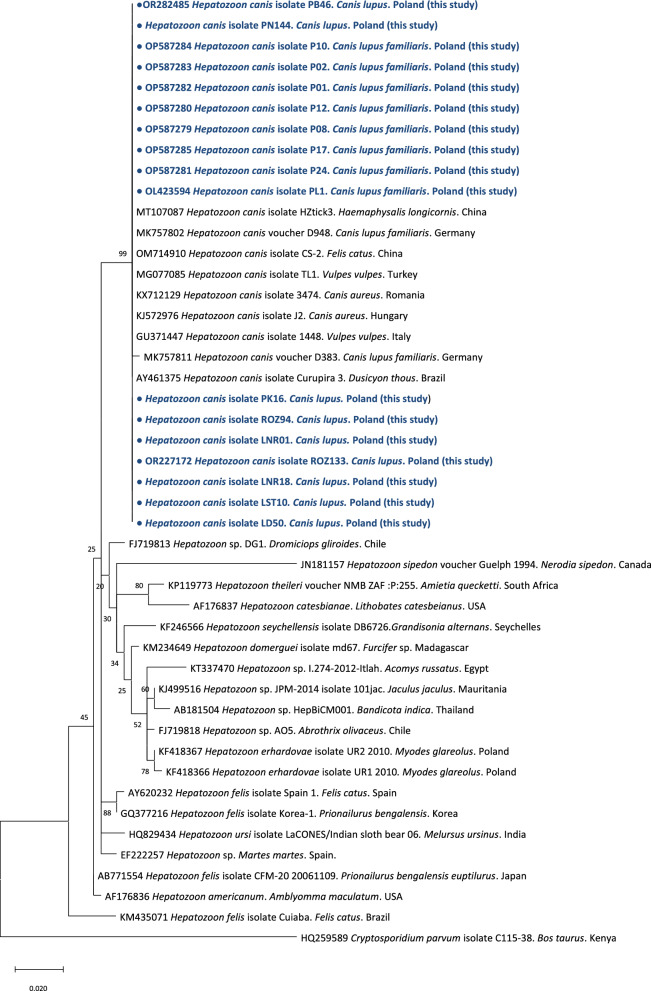
Evolutionary history of *Hepatozoon* based on the fragment of the *18S* ribosomal RNA gene was inferred by using the maximum likelihood method and Tamura 3-parameter model [34]. The tree with the highest log likelihood (− 1031.63) is shown. The percentage of trees in which the associated taxa clustered together is shown next to the branches. Initial tree(s) for the heuristic search were obtained automatically by applying the neighbour-joining and BioNJ algorithms to a matrix of pairwise distances estimated using the Tamura 3 parameter model, and then selecting the topology with the superior log likelihood value. The rate variation model allowed for some sites to be evolutionarily invariable ([+ *I*], 64.45% sites). The tree is drawn to scale, with branch lengths measured in the number of substitutions per site. This analysis involved 46 nucleotide sequences. The codon positions included were 1st + 2nd + 3rd + Noncoding. All positions containing gaps and missing data were eliminated (complete deletion option). In the final dataset, there were 314 positions. The nucleotide sequence of *Cryptosporidium parvum* was used as an outgroup. Evolutionary analyses were conducted in MEGA v. 11 [33]

In total, 11 cases were identified in 2020–2022. All of these cases were confirmed by PCR and then by sequencing and phylogenetic analyses of the obtained sequences (Fig. 3). Microscopic examination of the blood smears revealed the presence of *Hepatozoon* gamonts in the neutrophils of dogs (Fig. 2). In two dogs, parasitaemia was > 50%; in five dogs, parasitaemia was < 10%; and in four dogs, no gamonts could be found despite the positive results of the PCR tests (Table 2).

In almost half of the cases (*n* = 5), including the dog positive for *B. vulpes*, anaemia was diagnosed based on haematological testing (Tables 1, 2). Interestingly, the most common clinical signs associated with *H. canis* infection, namely lethargy, inappetence, cachexia and anaemia, were reported in five elderly dogs age > 10 years (Table 1). Younger dogs and puppies presented no clinical signs of hepatozoonosis, despite the presence of some haematological alterations (Table 2). Cases were identified among dogs living in rural areas (*n* = 3) and in stray (*n* = 4) or shelter (*n* = 4) dogs with no known history of foreign travel, indicating the likelihood of autochthonous infection.

### Wolves

DNA of *H. canis* was identified in 35% (21/60) of samples from 13 different regions/forest complexes of Poland (Fig. 1). Infection was found in 20 out of 57 blood samples and in one of three faecal samples. Information on animal sex was available for 56 animals (22 males and 34 females). Infection with *H. canis* was found in 29.4% (95% CL 17.7–44.2%) of females and 40.9% (95% CL 22.2–61.7%) of males (*t* = 0.8777, *df* = 54, *P* = 0.3840). Information on age was available for 51 animals (14 juvenile and 37 adult wolves). There was no difference in prevalence of *H. canis* between juvenile and adult wolves (35.7% [95% CL 15.3–62.9%] and 37.8% [95% CL 24.2–53.4%], respectively: *t* = 0.1372, *df* = 49,* P* = 0.8914).

### Genetic and phylogenetic analysis

Sequences obtained from 19 animals (10 from dogs, 9 from wolves) were almost identical, differing by only 1–3 nucleotides, revealing 99% identity to sequences of *H. canis* from the GenBank database obtained from wolves, golden jackals and foxes from Western and Central Europe (Fig. 3) [6, 12, 13, 22, 35, 36]. Sequences obtained from dogs were deposited in the GenBank database under accession numbers OL423594, OP587279 and OP587280–OP587285. Selected sequences obtained from wolves were deposited in the GenBank database under accession numbers OR227172 and OR282485.

## Discussion

In this study, we describe and confirm the first autochthonous *H. canis* infection in dogs and wolves from Poland.

In the last 10 years, there has been a growing number of reports of *H. canis* infection in carnivores in Central Europe, with hepatozoonosis reported in dogs in a number of countries neighbouring Poland: Ukraine [11], the Czech Republic [12], Slovakia [20] and Germany [13]. However, no cases were reported in dogs from Poland [24]. In a large epidemiological study carried out from 2003 to 2004 in 408 dogs attending veterinary practices, no dogs tested positive for *H. canis* [25]. In another study carried our a few years later (2009–2010), no *H. canis* DNA was identified among 126 sled dogs [24]. The first infected dog is reported in this study as case 9, and *H. canis* was retrospectively identified in a dog with clinical signs suspected of babesiosis in 2020. The series of cases described in the present study suggest that hepatozoonosis may become a growing problem in Poland: (i) the number of infected dogs increased in 2022; and (ii) it would appear that many hepatozoonosis cases may not have been recognised due to the lack of appropriate diagnostic testing used by veterinarians.

Hepatozoonosis and babesiosis can have similar clinical presentations in some animals, although *H. canis* infection is often subclinical. The nutritional and immunological status of each infected individual as well as co-infections and age may influence the course of infection [2]. In typical cases of canine babesiosis, moderate to severe anaemia (often haemolytic), thrombocytopenia, leukopenia with relative monocytosis and significant activation of mononuclear white blood cells are observed. In hepatozoonosis, haematological changes are less pronounced, although there is a potential for mild nonregenerative anaemia, leucocytosis with neutrophilia and monocytosis or mild thrombocytopenia [37]. Severe clinical manifestations (e.g. lethargy, fever, anorexia, weight loss, lymphadenomegaly and anaemia) are usually associated with a high parasite load [2, 38] or co-infection with other canine vector-borne pathogens [39, 40]. Both diseases can lead to elevated liver enzyme levels, but animals with hepatozoonosis more commonly present with increased total protein levels and polyclonal hyperglobulinaemia and hypoalbuminaemia.

In Poland, as in other countries, the prevalence of *H. canis* in free-living carnivores, especially red foxes, but also wolves, as reported in this study, is much higher than that in dogs [16, 19], which raises the question of the significance of the wild reservoir and the possible routes of infection. *Hepatozoon canis* would appear to be very efficient in infecting red foxes or grey wolves based on the prevalence of infection in these animals usually being > 50% and possibly even reaching 100% [12, 13, 23, 41]. Furthermore, infection in free-living carnivores is usually reported before the first cases are found in dogs [10, 16, 18, 21]. Thus, this kind of spillover is suspected to occur between free-living and domestic carnivores [10, 41]. The exact route of transmission is unknown. Protists of the genus *Hepatozoon* are generally vector-borne parasites [1, 42], but they are transmitted through the ingestion of an infected vector, not through a vector bite. *Rhipicephalus sanguineus *s.l. ticks have been shown to be competent vectors of *H. canis* in experimental studies [1]. Additionally, the ingestion of infected prey was confirmed as the route of infection for snakes or for *Hepatozoon americanum* in dogs [43]. Among the host population, the vertical path of infection, from mother to offspring, may be significant, as was proven for *H. canis* in dogs [44] or *Hepatozoon erhardovae* in voles [31]. Interestingly, we had three *H. canis*-positive puppies from one location, but we cannot confirm whether they were siblings born from an infected mother.

In the absence of *R. sanguineus* in Central Europe, there are two possibilities for infection: (1) *H. canis* is vectored by other tick species that commonly feed on wild carnivores and dogs or (ii) alternative routes of infection are of significance. At present, there is insufficient evidence for the vector role of other tick species: *H. canis* DNA was mostly identified in ticks feeding on possible infected hosts, such as foxes, jackals or dogs [21, 41]. Comparative analysis of *H. canis* in *Ixoides ricinus* and *R. sanguineus* nymphs collected from the same naturally infected dog revealed that sporogony occurred in *R. sanguineus* but not in *I. ricinus* [45]. The genetic material of *H. canis* has only rarely been found in questing ticks (< 0.1% of *I. ricinus*), thus not satisfying the conditions for efficient transmission [46]. *Hepatozoon canis* DNA was also found recently in 16 ticks (*Ixoides canisuga* and *Ixoides hexagonus*) collected from uninfected foxes [41], but experimental studies are still needed to confirm the vector role of these two tick species.

We hypothesise that the spillover of *H. canis* could have resulted from a horizontal route: the ingestion of infected fox meat. Stray and feral dogs are known to scavenge on road-killed animals or cadavers left by hunters [47, 48], while wolves actively hunt small carnivores [49, 50]. Rodents are not hosts for *H. canis* [31, 41, 51] so the predator–prey route could have been replaced with the scavenger behaviour of dogs. The horizontal transmission and this reservoir role of foxes are also supported by the fact that in an epidemiological study in Hungary, the occurrence of hepatozoonosis in dogs was significantly higher west of the Danube (vs. east of the Danube). In the western region, more fox and golden jackal infections occurred, while *R. sanguineus* s.l. was absent [10]. In many countries, for example in Germany, the Czech Republic and Slovakia, the high prevalence of *H. canis* in wildlife precedes the occurrence of hepatozoonosis in dogs. On the other hand, no local cases of hepatozoonosis were diagnosed in the Netherlands or Belgium, where the prevalence of *H. canis* in foxes and wolves is high [41]. In summary, the role of tick vectors in Central Europe seems of lesser importance than the vertical route of transmission (enabling the efficient spread of infection among free-living canids) and possible horizontal transmission by scavenger behaviour, allowing spillover to domestic animals.

Microscopic examination of the blood smears revealed the presence of *Hepatozoon* gamonts in the neutrophils of dogs with parasitaemia ranging from a few percent up to 50% (Table 2). A subclinical infection to mild disease is usually associated with low parasitaemia (1–5%), while severe infection can be found in dogs with high parasitaemia, occasionally approaching 100% of the peripheral blood neutrophils [1]. High parasitaemia rates can also be accompanied by extreme neutrophilia, reaching 150,000 leukocytes/l blood [1, 38, 52, 53]. In the present study, *H. canis* infections with high parasitaemia could have contributed significantly to the poor condition of two infected dogs (Tables 1, 2). However, co-infections with other vector-borne pathogens (*B. vulpes, D. repens*) could have aggravated the clinical status and outcome of these animals. Animals with low parasitaemia showed no signs of infection even if blood parameters (e.g., red blood cell and white blood cell counts) were not within the limits of the normal range.

The prevalence of *H. canis* in foxes in Poland has increased considerably in recent years [16, 19], leading to the expectation that there will likely be an increase in the number of new cases in dogs and wild canids. The recent rapid expansion of golden jackals from Mediterranean regions to Central and Northern Europe has also probably contributed to the rapid spread of this parasite to Central Europe [54]. As wild canids may be crucial for identifying potentially pathogenic emerging vector-borne diseases, further parasitological monitoring of their populations is highly recommended [55]. Our results support those reported previously, proving that monitoring for blood protozoan parasites (*Babesia* sp., *Hepatozoon* sp.) can be based on screening not only blood samples but also noninvasively obtained faecal samples [56, 57].

Our results provide information on *H. canis* prevalence based on 47 dog and 60 wolf samples. While those numbers may seem small in terms of epidemiological studies, this is the first report of *H. canis* infection in dogs in Poland, and this study provides initial information on the prevalence of *H. canis* in dogs and wolves in Poland. For this reason, the information reported here is fundamental for further research on this new pathogen in this country and is especially important for both veterinary practitioners and scientists.

## Conclusions

We present the first report of *H. canis* infection in dogs and wolves in Poland. Although the exact vector of this parasite is not known, veterinary practitioners should be aware of this new parasitosis and should consider appropriate diagnostics to confirm/exclude this infection.

Further studies need to be performed to understand the transmission routes of *H. canis* in domestic and wild canids in Poland.

## Data Availability

All relevant data are included in the article. Representative sequences obtained from dogs were deposited in the GenBank database under accession numbers OL423594, OP587279, and OP587280–OP587285. Selected sequences obtained from wolves were deposited in the GenBank database under accession numbers OR227172 and OR282485.

## Abbreviations

AL: Lysis buffer
ALP: Alkaline phosphatase activity
ALT: alanine aminotransferase
AST: Aspartate aminotransferase
CL: 95% confidence limits
HCT: Haematocrit
HGB: Haemoglobin
rRNA: Ribosomal ribonucleic acid
